# Regulation of Inflammatory Functions of Macrophages and T Lymphocytes by NFAT5

**DOI:** 10.3389/fimmu.2019.00535

**Published:** 2019-03-20

**Authors:** Jose Aramburu, Cristina López-Rodríguez

**Affiliations:** Immunology Unit, Department of Experimental and Health Sciences, Universitat Pompeu Fabra, Barcelona, Spain

**Keywords:** NFAT5/TonEBP, macrophages, T lymphocytes, osmotic stress, toll-like receptors, inflammation

## Abstract

The transcription factor NFAT5, also known as TonEBP, belongs to the family of Rel homology domain-containing factors, which comprises the NF-κB proteins and the calcineurin-dependent NFAT1 to NFAT4. NFAT5 shares several structural and functional features with other Rel-family factors, for instance it recognizes DNA elements with the same core sequence as those bound by NFAT1 to 4, and like NF-κB it responds to Toll-like receptors (TLR) and activates macrophage responses to microbial products. On the other hand, NFAT5 is quite unique among Rel-family factors as it can be activated by hyperosmotic stress caused by elevated concentrations of extracellular sodium ions. NFAT5 regulates specific genes but also others that are inducible by NF-κB and NFAT1 to 4. The ability of NFAT5 to do so in response to hypertonicity, microbial products, and inflammatory stimuli may extend the capabilities of immune cells to mount effective anti-pathogen responses in diverse microenvironment and signaling conditions. Recent studies identifying osmostress-dependent and -independent functions of NFAT5 have broadened our understanding of how NFAT5 may modulate immune function. In this review we focus on the role of NFAT5 in macrophages and T cells in different contexts, discussing findings from *in vivo* mouse models of NFAT5 deficiency and reviewing current knowledge on its mechanisms of regulation. Finally, we propose several questions for future research.

## Introduction

The nuclear factor of activated T cells 5 (NFAT5), also known as tonicity-responsive enhancer binding protein (TonEBP), belongs to the family of Rel homology domain (RHD) proteins (Rel family), whose other members are the NF-κB factors and the four calcineurin-activated NFAT1 to 4, also known as NFATc proteins (1–4). Since its molecular cloning 20 years ago (5, 6), NFAT5 has emerged as a relevant regulator of immune responses in diverse contexts.

The DNA-binding domain (DBD) is the only part of NFAT5 that resembles other Rel family proteins and apart from it, NFAT5 differs completely from NFATc and NF-κB proteins in its amino and carboxy-terminal regions where its transactivation domains reside (5–9). NFAT5 recognizes DNA elements with a [A/G]TGGAAA[C/A/T] sequence, which contains the characteristic GGAA core bound by NFATc proteins (6, 10), but lacks the characteristic calcineurin-regulated amino terminal domain that controls the nucleocytoplasmic shuttling of NFATc proteins. Indeed, part of the intracellular NFAT5 pool can be found in the nucleus of multiple cell types in basal steady state conditions independently of calcineurin (6), and can be constitutively bound to several gene promoters and enhancers in unstimulated mouse macrophages (11, 12).

As done with many other proteins, analysis of NFAT5 functions in primary cells has relied on the use of genetically modified mice deficient in NFAT5. Three main models have been described: (i) mice with a genomic deletion of the first (13) or first and second exons (14) of its DBD, which we refer to as NFAT5-null mice; (ii) NFAT5 heterozygous or haploinsufficient mice, carrying one intact allele that expresses a complete NFAT5 protein, and a deleted *Nfat5* allele lacking the first and second DBD-encoding exons (14); and (iii) conditional NFAT5-deficient mice in which NFAT5 is suppressed in specific cell lineages or in multiple tissues by crossing cell type-specific Cre recombinase transgenic (e.g., CD4-Cre, LysM-Cre, Mx-Cre, UBC-Cre/ERT2) mice with animals with both *Nfat5* alleles having *loxP* sites flanking the first DBD-encoding exon (floxed *Nfat5*) (15–18) (Table 1). Quite usefully, deletion of a genomic region of *Nfat5* encoding for a portion of its DBD suffices to cancel any mature NFAT5 protein product, as shown in T cells and macrophages both in NFAT5-null mice as well as in conditional, cell-specific NFAT5 deficiency models (16, 18) (Table 1).

**Table 1:** Immunological characteristics of NFAT5 deficiency mouse models.

**NFAT5 deficiency mouse model (References)**	**Loss of NFAT5 protein expression confirmed (References)**	**Cell types analyzed**	**Immunological effect of NFAT5 deficiency (References)**
Genomic deletion of the first exon of the DBD. NFAT5-null mice suffer severe perinatal mortality but 30% of mice survive to adulthood in a 129 background, whereas much fewer or no adult mice are obtained in a C57BL/6 background (13).	Essentially complete loss of NFAT5 protein confirmed by Western blot in mature T cells (16) and BMDM (11).	T lymphocytes, BMDM, tissue macrophages	Poor T cell responsiveness and function under high salt stress *in vivo* and *in vitro* (16). Altered balance of naïve and memory CD4 and CD8 T cells *in vivo* under high salt stress (16). *In vivo* defective rejection of allogeneic tumors (16). T cell proliferative deficiency under high salt (which *in vivo* is associated with systemic hypernatremia in these mice) (16). Defective response of BMDM to TLR stimulation *in vitro* in the absence of osmostress (11). *In vivo* defective expression of iNOS and impaired clearance of pathogen *Leishmania major* (11). Alterations in cytokine and TLR-regulated M1 and M2 polarization of BMDM *in vitro* (18). Reduced expression of CIITA and MHCII in macrophages (BMDM) (12).
Transgenic mice expressing a dominant-negative NFAT5 DBD in thymocytes and mature T cells under the control of a CD2 promoter (19).	Not applied	Thymocytes and mature T lymphocytes	Reduced numbers of thymocytes and mature T cells *in vivo*. Poor T cell responsiveness and function under high salt stress *in vitro*. Reduced T cell survival to amino acid deprivation in the absence of osmostress (19).
Genomic deletion of the first two exons of NFAT5 DBD. Severely impaired viability of NFAT5-null mice without reaching adulthood in a C57BL/6 background. Experiments in adult mice limited to heterozygous animals (14).	Western blot confirmed 50% reduction in expression of NFAT5 protein in thymocytes (14), peritoneal macrophages and BMDM of heterozygous mice with one mutant NFAT5 allele (20).	Thymocytes, mature T lymphocytes and BMDMs	Reduced numbers of thymocytes and splenocytes *in vivo* in heterozygous mice. Reduced Ig production upon immunization with OVA in heterozygous mice (14). Reduced proliferation in response to mitogenic stimuli for T (anti-CD3 and anti-CD28 antibody) and B cells (LPS) under high salt stress *in vitro* (14). Reduced T cell survival to amino acid deprivation in the absence of osmostress (14). NFAT5-haploinsufficient BMDM show poorer migratory capacity in response to M-CSF than wild-type ones (21). NFAT5-haploinsufficient peritoneal macrophages and BMDM show enhanced IL-10 expression in response to LPS than wild-type ones (20).
Systemic NFAT5 deletion upon tamoxifen administration in mice that have the first DBD exon floxed and are transgenic for a ubiquitin C (UBC) promoter-driven fusion of Cre/ERT2 activated by tamoxifen (17). These *Nfat5*-floxed mice have also been used to obtain NFAT5-deficient macrophages upon crossing them with LysM-Cre transgenic animals (22–24).	Essentially complete loss of NFAT5 protein in renal medulla (17) and 60–80% reduction of NFAT5 protein in BMDM in tamoxifen-treated mice (22). 50–80% reduction in NFAT5 protein confirmed by Western blot in BMDM from LysM-Cre x *Nfat5*^fl/fl^ mice (22).	BMDM, macrophages in footpad lesions by *Leishmania major* infection.	Enhanced susceptibility to infection with *Leishmania major in vitro* in NFAT5-deficient BMDM cultured from tamoxifen-treated UBC-Cre/ERT2 × *Nfat5*^fl/fl^ mice (22). *In vivo* defective expression of iNOS and impaired clearance of pathogen *Leishmania major* in footpad macrophages from LysM-Cre *Nfat5*^fl/fl^ mice kept in high salt diet (HSD) and infected with the parasite (22). Decreased local expression of VEGFC, and impaired lymphatic capillary density and chloride anion (Cl^−^) balance in skin of mice with a myeloid-specific deletion of NFAT5 (LysM-Cre *Nfat5*^fl/fl^) under HSD (23). *In vivo* reduced expression of iNOS and TNFα in peritoneal macrophages from LysM-Cre *Nfat5*^fl/fl^ mice injected with LPS (24).
Mice with the first DBD exon floxed allow deletion of NFAT5 upon crosses with lineage-specific Cre drivers (thymocytes and T cells Lck-Cre and CD4-Cre, myeloid LysM-Cre, blood lineages Vav-Cre), and the type I IFN-responsive driver Mx1-Cre (12, 15, 16, 18, 25).	Essentially complete loss of NFAT5 protein confirmed by Western blot in mature T cells (CD4-Cre x *Nfat5*^fl/fl^) (15, 16), thymocyte subsets (from DP to SP stages in CD4-Cre and Lck-Cre x *Nfat5*^fl/fl^) (25) and BMDM (Vav-Cre and poly I:C-treated Mx-Cre x *Nfat5*^fl/fl^) (18). Essentially complete loss of NFAT5 mRNA in peritoneal macrophages of LysM-Cre and Vav-Cre x *Nfat5*^fl/fl^ mice (18), and BMDM, BMDC, and lymph node and spleen T and B cells of poly I:C-treated Mx-Cre x *Nfat5*^fl/fl^ (12).	Thymocytes, T lymphocytes, BMDM, BMDC, tissue DCs and macrophages	Impaired proliferation of T cells, cell cycle arrest and defective induction of cell cycle regulators under high salt stress *in vitro* (15). Altered balance of naïve and memory CD4 and CD8 T cells and reduced homeostatic survival in response to IL-7 *in vitro* under high salt stress (16). Defective induction of CD24 in response to high salt stress *in vivo* and *in vitro* (16). Thymocyte development arrest at the transition from DN3 to DN4 associated with imbalanced expression of prosurvival and proapoptotic regulators (25). Defective induction of Th17 features in activated CD4 T cells in response to high salt (26). In addition, and independently of osmotic stress, activated CD4 T cells in CD4-Cre *Nfat5*^fl/fl^ conditional knockout mice show altered induction of Treg, Th1 and Th17 responses (26). Diverse osmostress-independent defects in macrophages: reduced activation by LPS, reduced phagocytic and bactericidal capacity against *Escherichia coli*, altered polarization responses to IFNγ plus LPS (M1) or IL-4 (M2), reduced capacity to stimulate Th1 T cell responses, and reduced anti-tumor activity in BMDM (18). Reduced expression of CIITA and MHCII in macrophages (BMDM, peritoneal, skin) due to lack of NFAT5 binding to a *Ciita* remote enhancer (12).

*NFAT5 knockout mice described in López-Rodríguez et al. (13), Go et al. (14), Küper et al. (17), all show substantial or complete loss of NFAT5 protein in different cell types analyzed, including T cells and macrophages. However, it has been found that mouse embryonic fibroblasts (MEFs) of NFAT5-deficient mouse models express a spliced NFAT5 product that lacks a portion of its DBD and is expressed at comparable protein levels to the wild-type NFAT5 protein (14, 24)*.

## Regulation of Macrophages and T Cells by NFAT5 Under Hypernatremia

NFAT5 was initially identified as a responder to hypertonic stress and inducer of an osmoprotective gene expression program in renal cells (5, 9), and so the first works on NFAT5 in immune responses focused on its function under high salt stress. The ability of leukocytes to respond to hypernatremia and express cytokines had already been reported in the 1990's (27–29), and early works on NFAT5 showed that it could induce TNFα and lymphotoxin-β in the human T cell line Jurkat cultured in high-sodium medium (30). Several studies have shown that the minimal level of hypernatremia at which immune cells (macrophages, T lymphocytes) activate NFAT5-dependent responses or exhibit functional defects if they lack this factor would be around 360–380 mOsm/kg, roughly equivalent to adding 30–40 mM extra sodium to a standard RPMI or DMEM culture medium. For instance, increasing medium osmolality from 300 mOsm/kg isotonic medium to 360 mOsm/kg with NaCl detectably enhanced the transcriptional activity of NFAT5 measured with reporter assays in mouse mitogen-activated primary T cells (31). NFAT5-dependent induction of endogenous osmostress-response genes in CD4 T lymphocytes was observed at 420 mOsm/kg (15) and reduced survival and proliferative capacity in NFAT5-deficient T lymphocytes were shown between 370 mOsm/kg and 420 mOsm/kg in independent studies (14, 15). This hypertonicity threshold was found to be similar in primary mouse bone marrow-derived macrophages (BMDM) and the mouse macrophage cell line RAW 264.7, in which activation of NFAT5 was observed upon addition of 40 to 65 mM NaCl to the culture medium (22, 31).

### Pathologic Systemic Hypernatremia

*In vivo*, levels of hypernatremia sufficient to activate NFAT5 have been observed in different conditions. Normal plasma tonicity in humans and common rodent models is around 290 mOsm/kg, with 128 to 140 mM Na^+^ (32, 33), but tonicity levels in the 340–400 mOsm/kg range have been recorded in the plasma of patients with osmoregulatory disorders (34–37), in aquaporin and vasopressin receptor-deficient mice (38–40), in dehydrated toddlers (41–43), and in hospitalized patients that developed hypernatremia (44, 45). Also, extensive burns can elevate plasma osmolality to 430 mOsm/kg (46). Intriguingly, NFAT5 itself can influence systemic tonicity and NFAT5-null mice were found to exhibit plasma hypernatremia and hyperosmolality (between 370 and 420 mOsm/kg) (16). This pathology is likely due to kidney dysfunction in NFAT5-deficient mice, which show atrophy of the renal medulla and impaired expression of genes that regulate osmotic homeostasis in the kidney (13). Constitutive plasma hypernatremia in NFAT5-deficient mice leads to lymphopenia and reduced ratio of naïve to effector T cells *in vivo* due to their defective adaptation to continued hypertonicity by lacking NFAT5 (16).

### Local Hypernatremia in Tissues

**Figure 1:**
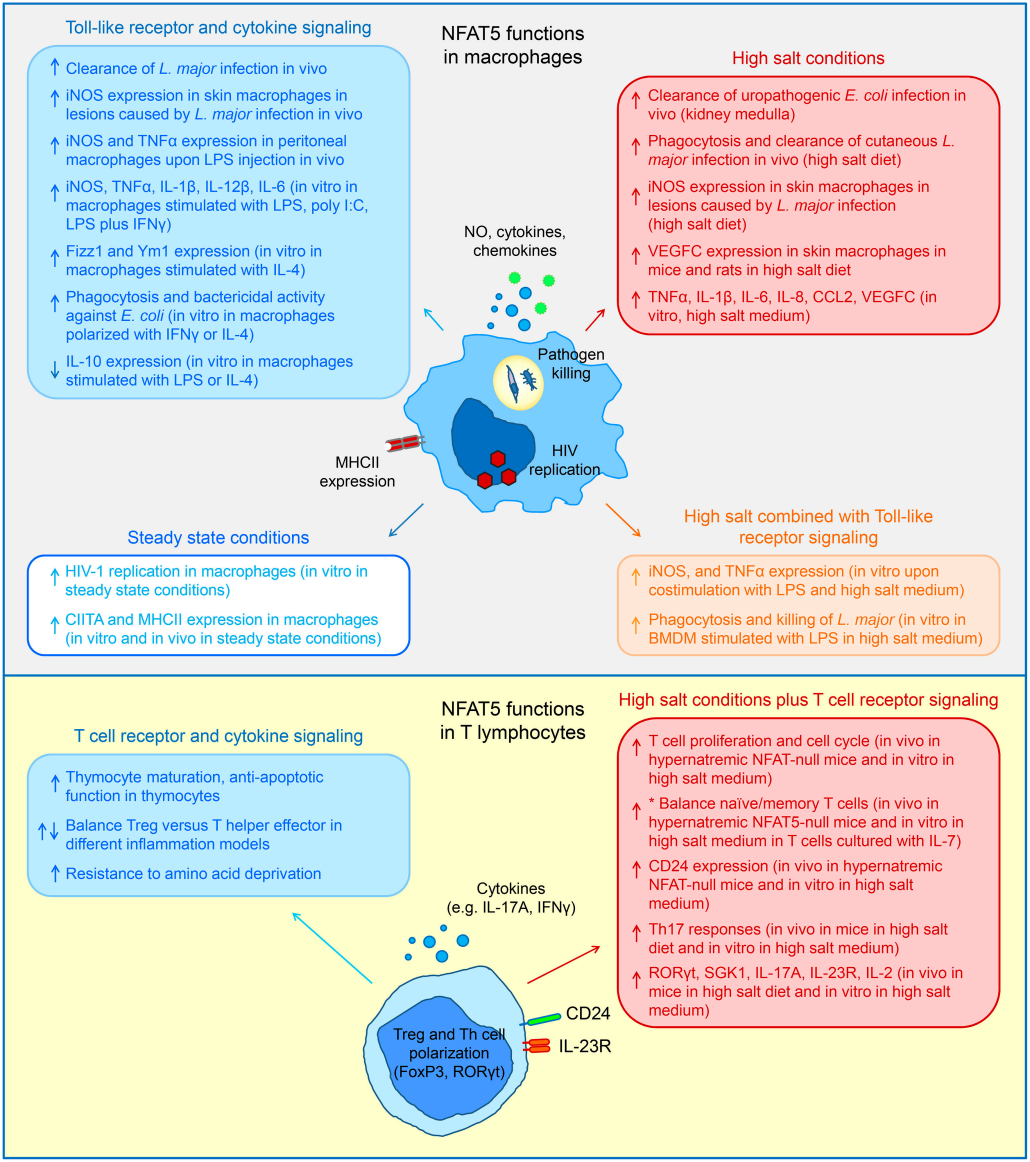
Schematic diagrams summarizing diverse functions of NFAT5 in macrophages and T lymphocytes in response to high salt conditions and signaling through different receptors (please see main text for more detailed description and references). Upward- and downward-pointing arrows indicate NFAT5-activated or –repressed functions respectively. ^*^The balance between naïve and memory T cells has been shown *in vivo* in steady state conditions in NFAT5-null mice and *in vitro* under conditions of homeostatic T cell maintenance with IL-7, not with T cell receptor simulation.

Apart from systemic plasma hypernatremia, local hypernatremia occurs naturally in the kidney medulla, where physiological tonicity of the interstitial fluid can be very high, 1,700 mOsm/kg with up to 690 mM sodium ion (Na^+^) as shown in laboratory hamsters (32). This microenvironment influences immune cells in the renal medulla including macrophages, dendritic cells (DCs) and T lymphocytes (47). The elevated hypertonicity of the renal medulla in humans and mice induces the production of CCL2 and CX3CL1 by resident epithelial cells in an NFAT5-dependent manner to attract infiltrating monocytes, which protect the kidney from genitourinary infections (48). Importantly, disruption of the renal sodium gradient or the CCL2-CCR2 axis or lack of NFAT5 in epithelial cells impairs monocyte recruitment. This defensive loop is amplified by the increase in phagocytic and bacterial killing capacity against *E. coli* and enhanced NFAT5-dependent inflammatory function (e.g., production of TNFα, IL-6, and IL-8) by infiltrating macrophages in response to high salt (48) (Figure 1). Other leukocyte populations in the renal medulla are also influenced by a local high salt environment. In mouse renal medullary DCs and bone marrow-derived dendritic cells (BMDCs), high salt promotes in DCs a phenotype that resembles alternatively activated or M2-like macrophages (49). In this regard, medullary DCs are poorer than cortical DCs at processing and presenting antigens via MHCII to T cells and are weak activators of intrarenal Th1 cells (50). By contrast, renal medullary DCs, as well as renal medullary CD14^+^ mononuclear phagocytes, are important effectors in innate immunity against uropathogenic *E. coli* and attract neutrophils to the medulla by secreting CXCL2 (48, 50). The role of NFAT5 in kidney DCs has not been explored yet.

Besides the continuously hypertonic milieu of the renal medulla, high sodium concentrations can occur in skin [see recent reviews by Titze et al. on the physiology of sodium accumulation in skin (33, 47, 51)]. ^23^Na magnetic resonance imaging (MRI) in infected skin wounds revealed localized elevation of ~40 mM of (Na^+^ + K^+^)-to-water ratio with respect to intact skin, and this was shown to enhance NFAT5-regulated macrophage antipathogen responses (22). In a different setting, rodents kept in a high salt diet (HSD) (4–8% NaCl diet plus 0.9–1% NaCl in drinking water for 2 weeks) exhibited elevated concentrations of sodium in skin from 154 mmol/kg baseline to 205 mmol/kg without increasing plasma sodium levels (23, 52, 53). This local hypertonicity caused skin mononuclear phagocytes to enhance secretion of VEGFC, which was transcriptionally induced by NFAT5. Locally secreted VEGFC in turn increased lymphatic capillary density with the effect of clearing excess chloride anion and ultimately buffering systemic blood pressure (23, 52, 53). This model provided important evidence that local NFAT5-dependent induction of VEGFC in immune cells in skin had a systemic impact in alleviating hypertension under HSD. On the other hand, DCs under hypernatremia seem to have the opposite effect and can promote hypertension in mice fed a high salt diet by producing IL-1β and immunogenic isolevuglandin (IsoLG) protein adducts that activate pro-inflammatory and pro-hypertensive T helper (Th)1 and Th17 cells (54). Whether NFAT5 is involved in pro-hypertensive mechanisms in DCs is not known yet. In parallel to its enhancing pro-inflammatory macrophage functions, high salt opposes M2 macrophage polarization, as shown by the decrease in M2 markers such as Mrc1/MMR and Fizz1 in skin and peritoneal macrophages from HSD-fed mice, *in vitro* in BMDM polarized to M2 with IL-4 and IL-13, and in human monocytes (55–58). The skewing of skin macrophage function toward inflammation under a high salt diet can have both beneficial and detrimental effects. On the one hand it can ameliorate cutaneous infection with *L. major* (22) but on the other, salt-induced attenuation of M2 markers *in vivo* is associated with poor healing of skin wounds (55). Of note, repression of macrophage M2 functions by high salt is not mediated by NFAT5 (55).

The pro-inflammatory effect of HSD is not limited to skin, and it can enhance the expression of pro-inflammatory macrophage markers such as iNOS and IL-6 in lung alveolar macrophages, both in basal conditions and in response to LPS inhalation (56) and in spleen macrophages (58). High salt diet has also been shown to be protective against vesicular stomatitis virus (VSV) and reduce viral burden in lung and brain, an effect attributed to enhanced induction of antiviral IFNβ by macrophages through the high salt-responsive factor ATF2/AP1 (57). High salt diet can also enhance macrophage inflammatory activation and infiltration in brain in a mouse model of experimental autoimmune encephalitis (EAE) (58). As we will see later, the exacerbating effect of HSD in EAE models also involves Th17 cells (56). Activation of macrophage inflammatory responses by high salt *in vivo* has thus been documented in diverse tissues including the renal medulla (48), skin in experimental models of high salt diet (23, 52) and lung epithelium in high salt diet or in response to seawater inhalation (56, 59). At least some of these functions are NFAT5-dependent. Intriguingly, high salt does not seem to influence in the same manner pro- and anti-inflammatory functions of macrophages and DCs. For instance, high salt promotes Arg1 expression in M2-like DCs but represses it in M2-polarized macrophages (49, 55). High salt also inhibits cross-priming capacity of dendritic cells to CD8 T cells, an effect that depends on the TLR signaling component TRIF but not on NFAT5 (60), a finding in line with the observation that DCs residing in the renal medulla are poor presenters of antigen to CD4 T cells (50). Further research is needed to understand how related innate immune cells like macrophages and DCs exhibit different sets of responses to high salt and specific roles of NFAT5 in them.

In T cells exposed to hypernatremia, NFAT5 helps maintaining proliferative capacity, homeostatic survival, and the balance of naïve and memory T cells (15, 16) (Table 1, Figure 1). Mitogen-activated mouse CD4 T cells lacking NFAT5 underwent cell cycle arrest together with an exacerbated DNA damage response in comparison with wild-type CD4 T cells when exposed to high salt (420–500 mOsm/kg) (15). Defective proliferative capacity of NFAT5-deficient T cells was also observed *in vivo* in experiments in which wild-type and NFAT5-deficient T cells were adoptively transferred in NFAT5-null host mice which suffer pathological hypernatremia (16). Further analysis identified the surface receptor CD24 as a relevant NFAT5-induced osmoprotective protein. CD24 is expressed at low levels in activated T cells, but is induced in an NFAT5-dependent manner under osmotic stress, even in moderate hypernatremia (380 mOsm/kg). Treatment with a CD24-blocking antibody inhibited the proliferative capacity of both CD4 and CD8 T cells in high salt medium (16).

Hypernatremia also promotes polarization of antigen-activated T lymphocytes toward Th17 cells in an NFAT5-dependent manner (26, 53). This effect involves the NFAT5-dependent induction of several pro-Th17 signaling and transcription regulators (Figure 1). Hypertonicity-activated NFAT5 induces the Th17-promoting kinase serum and glucocorticoid response kinase 1 (SGK1) (53), which in turn enhances the expression of the receptor of IL-23 (IL-23R) thus making T cells more responsive to IL-23-driven Th17 polarization (61). Mice on a HSD exhibited enhanced SGK1-dependent Th17 responses and exacerbated neurological damage in a model of EAE (53, 61). As shown in another work, exacerbated Th17 responses by HSD in the EAE model are also accompanied by the activation of inflammatory macrophages infiltrating the CNS (58). SGK1 was also found to skew the balance between T effector (Teff) and regulatory (Treg) cells toward enhanced effector and attenuated Treg responses in HSD-fed mice. These mice rejected heart allografts more strongly than animals on a normal diet, and this effect was associated with an SGK1-dependent increase in alloreactive CD4 T cell activation toward CD44^+^ effectors and reduced representation of FoxP3 regulatory T cells (62). Hypertonicity-activated NFAT5 also enhances the expression of the Th17 master transcription regulator RORγt, to whose promoter NFAT5 is recruited in mouse CD4 T cells exposed to hypernatremia, and induces IL-23R in a RORγt-dependent manner (26). By contrast, NFAT5 did not affect RORγt expression in Th17 cells induced with TFGβ and IL-6 in normal osmolality (26). NFAT5 was also recently shown to enhance expression of IL-2 mRNA in activated primary mouse CD4 T cells exposed to hypernatremia (26). IL-2 induction by high salt had been previously reported by the Jünger lab using Jurkat T cells activated with anti-CD3 plus anti-CD28 antibodies (63, 64). The stimulatory effect of high salt was mediated by release of intracellular ATP through the hemichannel pannexin-1 (Panx1) and subsequent activation of purinergic receptors P2X1, P2X4, and P2X7 by ATP released to the extracellular space. P2X signaling increases intracellular Ca^2+^ concentration and activates p38, which was needed for IL-2 induction (63, 64). Interestingly, T cells also release ATP through Panx1 during T cell receptor (TCR) stimulation in the absence of osmotic stress (65), and P2X receptors activate the calcineurin-dependent NFATc (65, 66) and promote the conversion of Treg to Th17 by reducing FoxP3 and enhancing RORγt expression (67). These results and the observation that osmostress-induced RORγt and IL-2 in T cells require simultaneous calcineurin activation (26) suggest that Th17 polarization under high salt might involve a cooperation between NFAT5-dependent mechanisms and other factors activated downstream P2X signaling.

## Osmostress-Independent Regulation of Macrophage Functions by NFAT5

### NFAT5 Regulates Macrophage Responses to Pathogen-Derived Products

The first observations that NFAT5 could regulate macrophage functions independently from its osmoresponsive activity came from the Goldfeld lab in 2006, who showed that NFAT5 enhances transcription from the HIV-1 long-terminal repeat (LTR) and replication of HIV-1 isolates in primary human monocyte-derived macrophages (68). Later research then uncovered that NFAT5 regulates the expression of multiple genes in primary mouse macrophages activated by Toll-like receptors (TLR). Recognition of pathogen-associated molecular patterns through TLRs by innate immune cells constitutes an important line of defense against microbial pathogens (69, 70). TLR signaling engages diverse transcription factors, including NF-κB, that promote the expression of hundreds of gene products with antimicrobial, inflammatory and immunostimulatory function (70, 71). Buxadé et al. found that NFAT5 induces multiple genes in primary mouse macrophages stimulated through different TLRs and TLR ligands, and in a manner independent from its osmostress response function (11) (Figure 1). NFAT5-mediated HIV-1 transcription was also found to be enhanced by TLRs in response to *Mycobacterium tuberculosis* (72). Macrophages lacking NFAT5 exhibited a more severe impairment in the expression of diverse TLR-induced gene products such as iNOS, TNFα, IL-6, and IL-12 when they were stimulated with low doses of TLR ligands, which suggests a relevant role in early stages of infection when pathogen burden may be relatively moderate. Correspondingly, NFAT5-null mice showed reduced ability to clear *Leishmania major* infections *in vivo*, and their skin macrophages exhibited poor induction of iNOS, which is needed to control parasite burden (11). Later studies showed that peritoneal macrophages from mice lacking NFAT5 in myeloid cells (LysM-Cre *Nfat5*^fl/fl^) also produced less iNOS and nitric oxide (NO) and lower levels of TNFα than wild-type mice in response to *in vivo* challenge with LPS (24). Buxadé et al. identified two modes of action for NFAT5, as this factor was found to be pre-bound to the promoters of several TLR-inducible genes such as *Tnf* and *Ccl2* already in steady state macrophages, but required TLR stimulation, IKKβ activity, and possibly histone acetyltransferase-dependent chromatin remodeling to be recruited to other genes such as *Nos2* and *Il12b* (11). TLR-induced recruitment of NFAT5 to the *Nos2* promoter or the *Il12b* enhancer were also found to depend on *de novo* protein synthesis, but not *de novo* synthesis of NFAT5 itself (11). This latter finding suggests that NFAT5 regulation by different signaling pathways might be mediated through the turnover of additional proteins, a possibility to be investigated in future works. The ability of NFAT5 to induce iNOS expression has also been shown in two non-immune cell types, SV40-immortalized human cardiomyocytes and HeLa cells (73). Qiu et al. described that tonic basal iNOS expression in these cells is sustained by NFAT5 and has a protective role against Coxackievirus infection, a major causing agent of viral myocarditis. Interestingly, hypertonicity enhanced iNOS expression in cardiomyocytes in an NFAT5-dependent manner and peritoneal administration of high salt solution to mice at the time of infection enhanced iNOS expression in the heart and had a protective antiviral effect (73).

Responsiveness of NFAT5 to TLRs and hypertonic stress enables macrophages to mount enhanced inflammatory responses when exposed to both types of stimuli simultaneously (Figure 1). This capacity has been documented in macrophages activated in skin wounds and thus sensing both a local increase in sodium and products from potential pathogens (22), and in the renal medulla during urinary tract infections, where a local high salt milieu enhances antibacterial defenses in an NFAT5-dependent manner (48). High salt also increases the capacity of LPS-stimulated macrophages to clear intracellular *Leishmania* parasites in an NFAT5-dependent manner (22), although NFAT5 can enhance antibacterial and phagocytic capacity of macrophages against *E. coli* in the absence of osmotic stress (18).

### NFAT5-Regulated Macrophage Responses in Inflammatory Pathology

The role of NFAT5 as promoter of proinflammatory macrophage function has been further documented in mouse models (21) as well as in studies in human rheumatoid arthritis (RA) patients (74). Halterman et al. showed that NFAT5 haploinsufficient mice crossed to ApoE-deficient mice developed less severe atherosclerosis under high fat diet than NFAT5-wild-type and ApoE-deficient mice (21). The authors tracked the pro-atherosclerosis effect of NFAT5 to bone marrow-derived cells, suggesting that the cells responsible were of hematopoietic origin, although its specific type was not identified. Another work using the same NFAT5-haploinsufficient mouse model (14) showed that these animals developed less severe arthritis than wild-type ones in an experimental model of collagen-induced arthritis (75). Later work using a mouse model of arthritis induced by albumin plus IL-1β in NFAT5-haploinsufficient mice showed that NFAT5 and the NFAT5-induced chemokine CCL2 were positive contributors to the inflammatory pathology of RA in the arthritic lesion, also finding that CCL2 was a macrophage prosurvival factor (74). Direct infusion of macrophages in the joint of otherwise normal mice showed that NFAT5-deficient macrophages caused a less severe inflammatory pathology than wild-type ones, confirming that NFAT5-dependent regulation of macrophages was a key component in exacerbating joint inflammation (74). The same work reported that synovial macrophages from RA patients expressed more NFAT5 mRNA and protein than control macrophages from healthy individuals, which suggests that the mechanisms of inflammation in human patients and mouse models may similarly involve NFAT5.

### NFAT5 Influences Pro- and Anti-Inflammatory Macrophage Polarization

Altogether, different works from independent groups have documented the ability of NFAT5 to enhance pro-inflammatory responses in macrophages in different contexts. However, a recent work has shown that in mouse BMDM cultured with IL-4, which promotes the polarization of alternative macrophages (often referred to as M2 macrophages), generally associated with anti-inflammatory and tissue-repair functions, NFAT5 can enhance the expression of the IL-4-inducible gene products Fizz1 and Ym1, and to a lesser extent Arg1 (18). By contrast, NFAT5 partially repressed the expression of CD206, another marker associated with M2 macrophage polarization. Intriguingly, although Fizz1 (also known as Relmα or *Retnla*) and Arg1 are often used as markers of IL-4-polarized M2 macrophages, they have been shown to attenuate Th2 T cell responses and favor pro-inflammatory Th1 cells *in vivo* (76, 77). In this regard, Tellechea et al. found that T cells activated by anti-CD3 antibody and syngeneic BMDM induced poorer Th1 and stronger Th2 responses if BMDM lacked NFAT5. Adoptive transfer experiments of BMDM in mice harboring ID8-Luc ovarian tumors in the peritoneal cavity or subcutaneous Lewis lung carcinoma (LLC) tumors showed that NFAT5-deficient macrophages were less effective than wild-type ones at delaying tumor growth, an observation consistent with being less capable of maintaining a pro-inflammatory or M1 phenotype (18). However, tumors inoculated in mice that lacked NFAT5 in myeloid cells (LysM-Cre *Nfat5*^fl/fl^) grew the same as in wild-type mice, and both wild-type and NFAT5-deficient macrophages isolated from the peritoneum of mice with ID8-Luc tumors or from subcutaneous LLC tumors showed a marked M2-like phenotype. The different results between injecting a relatively large number of exogenous BMDM in the tumor or letting the tumor recruit endogenous macrophages as it expanded could suggest, among other interpretations, that although NFAT5 can favor the polarization of pro-inflammatory macrophages under appropriate stimulation, this influence cold be neutralized in a sustained pro-M2 microenvironment imposed by the tumor. Overall, results in several works agree on the general theme that NFAT5 contributes to sustain a tone of pro-inflammatory or pro-Th1 macrophage readiness (11, 18, 20). However, studies on the regulation of anti-inflammatory responses by NFAT5 have produced different results with different cell models and stimuli. For instance, both Tellechea et al. and Choi et al. showed that NFAT5 can attenuate the expression of CD206, but whereas Tellechea at al., reported that NFAT5 enhanced Arg1 induction in IL-4-stimulated BMDM (18), Choi et al. found that it repressed it in the mouse macrophage cell line RAW 264.7 and in human monocyte-derived macrophages stimulated with either LPS or IL-4 (20). Choi et al. also found that reduced NFAT5 expression in haploinsufficient peritoneal macrophages and BMDM led to enhanced IL-10 production upon LPS stimulation and showed that IL-10 enhanced Arg1 expression RAW 264.7 cells (20). These findings suggest that the positive or negative effect of NFAT5 on specific anti-inflammatory macrophage functions may differ in different contexts, and further work will be needed to elucidate these differences.

### NFAT5-Regulated MHCII Expression in Macrophages

Besides regulating macrophage function under pro- and anti-inflammatory stimuli, NFAT5 has been recently shown to sustain tonic expression of MHCII in mouse macrophages in homeostatic or steady state conditions without overt stimulation (12) (Figure 1). BMDM, peritoneal and skin resident macrophages lacking NFAT5 express reduced basal levels of MHCII mRNA and concomitantly MHCII surface expression, and cultures of macrophages lacking NFAT5 contained fewer cells expressing high levels of surface MHCII than wild-type cells. In line with these findings, NFAT5-deficient BMDMs were poorer stimulators of MHCII-dependent responses in T lymphocytes than wild-type macrophages. Also, skin grafts of male conditional knockout mice that lacked NFAT5 in myeloid cells, and which had fewer MHCII^+^ macrophages than wild-type skin controls, were rejected less rapidly than wild-type grafts when transplanted in female recipients. Defective MHCII expression in macrophages that lacked NFAT5 could be overcome by stimulating them with IFNγ, a potent MHCII inducer, but then again NFAT5-deficient macrophages rapidly decreased MHCII expression when IFNγ was removed. This work found that the mechanism by which NFAT5 sustains MHCII expression is through the MHCII transactivator CIITA, whose expression is regulated by NFAT5 binding to and activating a remote enhancer 49 kb upstream of *Ciita* transcription start site. Buxadé et al. found that in native chromatin this enhancer is juxtaposed with the *Ciita* promoter I, which directs CIITA expression in myeloid cells, and this three-dimensional enhancer-promoter configuration is NFAT5-dependent (12). The authors also analyzed whether NFAT5 regulated MHCII in DCs. DCs are closely related to macrophages, express MHCII in a CIITA-dependent manner, and also transcribe *Ciita* from promoter I. However, lack of NFAT5 in DCs did not affect expression of CIITA nor MHCII, and moreover DCs did not recruit NFAT5 to the−49 kb site nor exhibited the three-dimensional loop between it and the promoter I of *Ciita*. At present we know much less about the role of NFAT5 in DCs and other myeloid cells than in macrophages, but the findings in Buxadé et al. suggest that they could use NFAT5 to different effects. One can expect that future research will uncover patterns of common and cell-specific functions regulated by NFAT5 in different immune cell lineages throughout their ontogeny.

## Osmostress-Independent Regulation of T Lymphocytes by NFAT5

### NFAT5 Regulates Thymocyte Ontogeny and Mature T Lymphocyte Function

The first evidence of NFAT5 regulating T lymphocytes in the absence of osmotic stress came from the analysis of transgenic mice that expressed a T cell-restricted NFAT5 DBD (19). This domain acts as a dominant negative for NFAT5 as it competes for DNA binding and also displaces the endogenous dimers of NFAT5 (30). This DBD also gets progressively accumulated in a DNA-bound pool after successive rounds of cell division and thus may block access of endogenous NFAT5 to chromatin in a durable manner (78). NFAT5 DBD-transgenic mice had normal CD4 cellularity but fewer CD8 T cells in spleen and lymph nodes than non-transgenic controls. Transgenic CD8 T cells responded normally in proliferation and cytokine (IL-2, IFNγ, IL-4) production assays upon stimulation with anti-CD3 and anti-CD28 antibodies or mitogens, but exhibited reduced viability upon prolonged culture, which was associated with poorer survival to amino acid depletion (19). This observation raises the possibility that NFAT5 could confer survival advantages to T cells in situations where they could find themselves under reduced nutrient availability, such as inside tumors, in inflamed lesions or ischemic tissues. This interesting possibility remains an open question for future studies.

More recently, other works using T cell-conditional NFAT5-knockout models have identified NFAT5 functions in thymocytes and mature T cells (25, 26, 79) (Figure 1). NFAT5 is expressed in developing thymocytes from the CD4- and CD8-double negative (DN) stage to single positive CD4 and CD8 mature thymocytes (25). NFAT5 expression in DN thymocytes is sustained by the pre-TCR and IKKβ signaling, and deleting NFAT5 at this stage causes defective transition from DN3 to DN4 stages, altered β-chain allelic exclusion and increased apoptosis in DN and double positive (DP) thymocytes (25). NFAT5-deficient thymocytes were also found to have an imbalance in apoptosis regulators, with reduced expression of prosurvival factors A1 and Bcl2 and increased activity of the proapoptotic p53/Noxa axis. These defects in thymic T cell ontogeny were then reflected in a reduced number of CD4 and CD8 double positive (DP) thymocytes and peripheral CD4 and CD8 mature T cells (25). Importantly, NFAT5-deficient thymocytes expressed NFAT5-regulated tonicity response markers *in vivo* to the same level as wild-type thymocytes, arguing that the physiological thymus microenvironment does not represent a hypertonic niche nor elicits an NFAT5-dependent osmostress response. Interestingly, deletion of NFAT5 at the later DP stage did not cause obvious defects in the maturation of single positive CD4 and CD8 thymocytes and in the cellularity of peripheral mature CD4 and CD8 lymphocytes (25). However, although loss of NFAT5 in DP thymocytes caused no obvious dysfunction in their descendant mature CD4 T cells in homeostasis, these exhibited altered responses to pro-inflammatory stimulation (26). NFAT5-deficient CD4 T cells activated *in vivo* with anti-CD3 antibody exhibited a mild bias toward reduced induction of Foxp3^+^ CTLA4^+^ cells and higher proportion of IFNγ-expressing cells, and *in vitro* Th1 polarization assays showed that IFNγ expression was more prolonged in NFAT5-deficient CD4 T cells than in wild-type ones. These findings suggested that NFAT5 can attenuate excessive pro-inflammatory responses in T cells by either promoting Tregs or decreasing Th1 activation. Notably, the pattern of NFAT5-regulated cytokines in CD4 T cells differed between normal tonicity (300 mOsm/kg) and high salt (420 mOsm/kg). For instance, NFAT5 enhanced IL-2 induction only when T cells were activated by anti-CD3 plus anti-CD28 antibodies in high salt, but did not affect it during stimulation in isotonic conditions. NFAT5 also enhanced the Th17 markers RORγt and IL-23R in response to high salt but was neutral when T cells were activated *in vitro* in the presence of Th17-polarizing cytokines TGFβ plus IL-6 without osmotic stress. Then, NFAT5 attenuated acute IFNγ responses while enhancing FoxP3 and CTL4-expressing CD4 Treg cells in mice injected with anti-CD3 antibody, but neither IFNγ nor FoxP3 were sensitive to NFAT5 in CD4 T cells stimulated in culture in high salt medium.

### NFAT5-Regulated T Cell Function in Inflammatory Pathology

In line with the finding that NFAT5 can attenuate Th1 responses, Alberdi et al. showed that mice lacking NFAT5 in T cells developed exacerbated colitis in response to dextran sodium sulfate (DSS), associated with enhanced levels of IFNγ and IL-17A mRNA in colon lesions as well as increased IFNγ and IL-17A mRNA and pro-inflammatory macrophage markers in mesenteric lymph nodes (26). These results were consistent with an earlier work that supported NFAT5 contribution to Treg function (80). Li et al. described that a 50% reduction of NFAT5 expression with siRNA in human CD4 T cell cultures caused a mild attenuation in the expansion of Foxp3^+^ CD25^+^ CD4 Tregs and a mild decrease in their production of the Treg-associated cytokine TGFβ1 (80). The same work identified miRNA-568 as a repressor of NFAT5 expression and Treg function in CD4 T cells (80). However, a recent work by Serr et al. showed that T cells isolated from mice made to delete NFAT5 systemically induced more FoxP3^+^ Treg cells in response to CD3 plus CD28 stimulation than wild-type lymphocytes (79). The Treg bias was associated with enhanced expression of the lipid phosphatase PTEN in NFAT5-deficient T cells. The authors also found that NFAT5 expression in T cells was increased by miRNA-181A, which was positively associated with exacerbated autoimmunity in type-1 diabetes in humans and mouse experimental models (79). Then Serr et al. tested the potential contribution of NFAT5 to autoimmune diabetes in mice using an antagomir to block miRNA-181A and an NFAT5 chemical inhibitor KRN5 (79). Both approaches enhanced Treg responses, a finding consistent with NFAT5 attenuating Treg function. However, it is also possible that some of the effects of miRNA-181A and the KRN5 inhibitor could be independent of NFAT5. For instance, miRNA-181A not only enhances the expression of NFAT5 in T cells but also NFAT1/c2 and NFAT4/c3 (79), which can enhance T cell pro-inflammatory polarization, and the KRN5 inhibitor does not inhibit NFAT5 directly but works by blocking the binding of NF-κB to the *Nfat5* promoter in macrophages and thus reducing NFAT5 expression (81). While macrophages treated with this drug express less of several pro-inflammatory gene products that are NFAT5-regulated (11), the same genes are also NF-κB targets. Thus, it is possible that the ability of miRNA-181A antagonists and the KRN5 inhibitor to attenuate autoimmune type-1 diabetes could result from combined effects on NFAT5, NF-κB, and NFATc factors. In this regard it would be informative to test this synthetic inhibitor in NFAT5-deficient cells to assess NFAT5-independent effects that might be attributable to NF-κB. Nonetheless, and focusing just on results obtained with NFAT5-deficient T cells, the studies by Alberdi et al. and Serr et al. suggest that NFAT5 may not have a fixed function in T cells, in this case the Treg to T effector balance, but rather modulate their activity in different directions depending on their polarization and activation context. Here it is interesting to observe that while Alberdi et al. used mice that lacked NFAT5 only in mature T lymphocytes, albeit permanently through life, Serr et al. used mice in which NFAT5 was deleted systemically upon administration of tamoxifen in adult animals and thus went from expressing NFAT5 normally to lose it in multiple cell types. It seems likely that the functional repertoires of T cells and how they communicate with normal or NFAT5-deficient macrophages and other cells will be different in both models. Future analyses comparing mouse models where NFAT5 has been deleted in particular subsets of immune cells with systemic NFAT5 deletion models will help to gain a deeper understanding of NFAT5 roles in immune responses.

In this context it is worth discussing the only human patient with an NFAT5 deficiency characterized so far. Boland et al. identified an individual diagnosed with autoimmune enterocolopathy with symptoms resembling inflammatory bowel disease, and whose T lymphocytes expressed NFAT5 to about 20% of the level of healthy individuals (82). The patient had normal plasma osmolality. *In vitro* assays with this patient's T cells showed that they had reduced resistance to high salt stress, as expected from their NFAT5 deficiency, but also exhibited altered function in the absence of osmotic stress. The patient's CD4 T cells induced TNFα and IFNγ normally after short-term unpolarized stimulation, but his CD8 T cells displayed reduced production of both cytokines (82). Boland's study did not analyze Treg function, but found that the patient had fewer NK cells and an altered distribution of B cell subsets with respect to healthy individuals, suggesting that insufficient expression of NFAT5 had an impact on diverse leukocyte populations. The authors also observed that patients with ulcerative colitis and Crohn's disease had lower expression of NFAT5 mRNA in intestinal tissue than healthy controls (82). Altogether, findings by Boland et al. and Alberdi et al. support an association between reduced NFAT5 expression in T cells and enhanced intestinal inflammation. However, these studies addressed different scenarios of NFAT5 deficiency and intestinal inflammation, and the underlying causes of exacerbated inflammatory processes in both contexts might, although related to NFAT5, turn out to be mechanistically different.

## Signaling and Regulation of NFAT5

### Nuclear Translocation and Transcriptional Function of NFAT5

Our knowledge on signaling pathways regulating NFAT5 in immune cells is limited, but works in other cell types and lines, mainly kidney-derived cell lines and fibroblasts, have reported a number of kinases and phosphatases that can influence its nuclear accumulation and transcriptional activity [please see (83) for a recent review compiling current literature on NFAT5-regulating kinases and phosphatases]. Nuclear accumulation of NFAT5 in response to high salt is promoted by the DNA damage and ROS-responsive kinase ATM (84, 85), CDK5 (86), and by the tyrosine kinase c-Abl (87), but is opposed by CK1 (88). Transcriptional activity of NFAT5 in response to high salt, assessed by reporter assays or induction of NFAT5-dependent genes in kidney cell lines and fibroblasts, is promoted by ERK2 (89, 90) activated through upstream PKCα and PLCγ1 (90–92), by p38α (31, 93), FAK (94), ATM (85, 95), and mTORC1 (96). NFAT5 activity can be also stimulated by PKA, AKT1 and PI3K through their inhibition of the NFAT5-repressive kinase GSK-3β (97), and by the tyrosine kinases c-Abl (98, 99) and Fyn (100). As for NFAT5 repressors, the tyrosine phosphatase SHP-1 (99) and the kinases GSK-3β (97) and p38δ (93) have been found to attenuate NFAT5 activity. At least some of these pathways, such as p38, ATM, PI3K, and mTORC1 have been found to regulate the transcriptional activity of NFAT5 in T cells and macrophages in response to hypertonic stress (22, 26, 31, 96). These kinases also influence diverse immune responses independently from osmotic stress, yet their impact on NFAT5 in non-osmostress scenarios has not been much explored. For instance, inhibition of p38α reduced the transcriptional activity of NFAT5 in LPS-activated RAW 264.7 macrophage cells (101), but did not impair its LPS-induced recruitment to the *Nos2* promoter in primary BMDMs, which instead depended on IKKβ (11).

### Regulation of NFAT5 Protein Abundance in Macrophages and T Cells

Macrophages treated with high salt or stimulated with TLR ligands, and TCR-stimulated T lymphocytes exposed to high salt exhibit a progressive increase in NFAT5 abundance. Accumulation of NFAT5 in TLR-activated macrophages spans hours, requires sustained transcription and IKKβ activity, and involves a dynamic balance between synthesis and degradation (11). Expression of NFAT5 mRNA induced by TLR stimulation is promoted by an IKKβ-NF-κB-dependent transcriptional mechanism, as p65/NF-κB was recruited to the *Nfat5* promoter in a TLR-dependent manner, and TLR-enhanced NFAT5 expression was precluded by pharmacological inhibitors of IKKβ and in IKKβ-deficient macrophages (11). In macrophages stimulated with LPS and high salt, NFAT5 accumulation and NFAT5-dependent induction of iNOS are also enhanced by p38α (22). Inhibiting p38 also decreased NFAT5 expression in RAW 264.7 macrophage cell line stimulated with only LPS for 24 h (101), but in primary macrophages inhibition of p38 with SB203580 did not reduce long-term accumulation of NFAT5 upon LPS stimulation (also 24 h) nor short-term (2 h) LPS-induced recruitment of NFAT5 to the *Nos2* promoter (11). A role for p38 in NFAT5 accumulation induced by high salt has also been described in primary splenocytes and the human CD4 T cell line Jurkat. In both, suppressing p38α with siRNA or eliminating Brx, an osmosensing scaffold that together with Rac1 (91, 102) and OSM (91) activates MKK3 upstream p38, inhibited the accumulation of NFAT5 mRNA and protein induced by high salt (103). Here it is worth noticing that in macrophages responding to LPS the peak of induction for NFAT5-responsive genes such as *Nos2, Tnf*, *Il6*, and *Il12b* is reached between <1 h and 4 h, a time at which NFAT5 abundance has increased little with respect to basal levels. This suggests that preexisting levels of NFAT5 suffice to start a robust wave of induction of target genes, whereas its slow accumulation over time could play a more important role in sustaining cellular responses during extended periods of stimulation or stress conditions. NFAT5 abundance in T cells is also regulated by miRNAs miRNA-181A and miRNA-568 (79, 80). Other miRNAs have been identified as regulators of NFAT5 expression in renal medullary cells responding to high salt (104), so it is likely that regulation of NFAT5 by diverse miRNAs might be generally operational in different cell types and stimulation conditions.

### NFAT5 and Calcineurin

Activation of T cells by TCR signaling engages the phosphatase calcineurin, a major activator of NFATc proteins (3). As to whether NFAT5 can respond to calcineurin, it has been shown that calcineurin activation enhances the accumulation of NFAT5 protein in primary mouse T cells and the Jurkat T cell line upon stimulation with phorbol esters and the calcium ionophore ionomycin, whose combination roughly mimics signaling from the TCR (30, 105). How calcineurin signaling can enhance NFAT5 abundance is unknown, but it is possible that this effect could follow the general activation of the T cell upon TCR stimulation. In contrast to NFATc proteins whose nuclear translocation is rapidly activated by calcineurin-mediated dephosphorylation, calcineurin does not appear to enhance nuclear translocation of NFAT5 (30), and an early work showed that it did not cause its bulk dephosphorylation in T cells (6). Regarding NFAT5 transcriptional activity, one article showed that an NFAT5-responsive reporter could be activated by phorbol esters and ionomycin in a calcineurin-dependent manner in Jurkat T cells (105). However, another work, also with Jurkat T cells and using a dual NFATc and NFAT5-responsive reporter and peptides that selectively inhibited NFATc activation by calcineurin (106) or NFAT5 homodimerization (30), found that activation of the reporter by phorbol ester and ionomycin was due to calcineurin-activated NFATc proteins and not NFAT5 (31). On the other hand, calcineurin is needed for the expression of osmostress-inducible, NFAT5-regulated genes in mouse CD4 T cells activated through CD3 and CD28 in high-salt medium, arguing that calcineurin signaling influences the pattern of genes that NFAT5 can induce by osmostress (26). Altogether, evidence suggests that calcineurin and NFAT5 communicate at some level, but at present it is unclear how calcineurin affects NFAT5 activity and whether it may do so through specific posttranslational modifications.

### NFAT5 Responsiveness to Hypertonicity in T Lymphocytes Requires Their Prior Activation

Another aspect of NFAT5 transcriptional activity in which macrophages and T cells differ is that NFAT5 can respond to high salt directly in macrophages, but its capacity to induce osmostress-response genes in T cells seems to require prior T cell activation. An early work using primary T cells transgenic for an NFAT-responsive luciferase reporter found that the hypertonicity threshold required for NFAT5-mediated activation of the reporter varied with the T cell activation state (31). This reporter did not respond to hypertonicity (380–400 mOsm/kg) in freshly isolated lymphocytes, showed a robust response in cells that had been activated with mitogens for 24 h, and became again less responsive in cells further cultured during 48 to 72 h. Also, NFAT5-dependent induction of the mRNAs of RORγt, IL-17A, IL-2, and the osmoprotective gene product aldose reductase (AR) in CD4 T cells stimulated through CD3 and CD28 in high salt medium was downregulated if calcineurin and mTORC1 were inhibited (26). Indeed, a positive role of mTORC1 enhancing osmostress adaptation and induction of osmoprotective genes, both through NFAT5-dependent and—independent mechanisms has been also described in mitogen activated splenic T cells and immortalized mouse embryonic fibroblasts (MEFs) (96). Altogether, these results indicate that signaling pathways such as calcineurin and mTORC1 activated by TCR and growth factors can enhance the responsiveness of T cells to high salt and influence the pattern of hypertonicity-induced genes. These pathways may differ in other immune cells, and for instance primary BMDM effectively activated NFAT5-dependent response to high salt (430 to 530 mOsm/kg) without needing simultaneous calcineurin activation (31). How T cell activation makes NFAT5 better able to induce certain genes in response to high salt is not well understood. On the one hand, NFAT5 abundance and transcriptional activity are higher in mouse CD4 T cells in S and G2 phases than in G1, which suggests that growing and proliferating T cells are better poised for inducing stronger NFAT5 responses (15). As for potential mechanisms underlying the stronger responsiveness of NFAT5 in activated T cells, several possibilities can be discussed. Enhanced signaling and biosynthetic capabilities of activated T cells (107, 108) might result in more robust signaling to NFAT5; also the general chromatin decondensation that occurs upon T cell activation could make potential NFAT5 target genes more accessible to its binding, as shown for the IL-2-activated STAT5 (109) and c-Myc (110). These or additional mechanistic possibilities could be addressed in future studies.

### Interactions Between NFAT5, AP-1, and NF-κB

Last, interactions between NFAT5, AP-1 (Fos/Jun), and NF-κB have been reported. The calcineurin-activated NFATc proteins NFAT1 through 4 are all capable of interacting with c-Fos/Jun (AP-1) dimers on composite DNA sites. This interaction involves 11 residues in the DBD of NFATc and requires that both NFAT and AP-1 bind to specific DNA sites (111). Early work on NFAT5 showed that its DBD lacks 8 of 11 residues that are conserved in NFATc and does not interact with Jun or c-Fos (6), and structural characterization of the DBD of NFAT5 showed that it binds DNA as a constitutive dimer whose conformation and orientation toward DNA does not support a simultaneous interaction with AP-1 (10). Other articles also reported that overexpression of a dominant negative NFAT5 DBD in Jurkat T cells did not interfere with the activation of composite NFAT/AP-1-responsive and AP-1-responsive reporters (19, 30). However, a later work showed that Jun and c-Fos coimmunoprecipitated with a tagged full-length recombinant NFAT5, as well as with its separate amino- and carboxy-terminal domains expressed in the human embryonic kidney-derived HEK293 cell line (112). These interactions were constitutive regardless of whether cells were in isotonic or hypertonic medium, and downregulation of AP-1 with siRNAs or mutation of AP-1 sites adjacent to NFAT5 binding sites in reporters reduced their activation by hypertonicity (112). These results suggest the possibility that NFAT5 might be capable of interacting with c-Fos and Jun proteins through contact residues and mechanisms different from those used by the calcineurin-activated NFATc, but so far its structural basis and functional relevance have remained unexplored. More recently, Roth et al. reported the cooperation of NFAT5 with NF-κB in the induction by hypertonic stress of the cytokines TNFα, MCP-1 (CCL2) and the osmoprotective gene product AR (113). This work showed that NFAT5 and p65/NF-κB could coimmunoprecipitate in mpkCCDc14 cortical collecting duct mouse cells exposed to hypertonic stress, and downregulating NFAT5 with siRNA reduced the activation by hypertonic stress of an NF-κB-driven reporter. However, earlier works had found that a dominant negative NFAT5 DBD did not interfere with the activation by phorbol esters and ionomycin of NF-κB-driven reporters in Jurkat T cells, while effectively inhibiting an osmostress-activated NFAT5 reporter (19, 30). From this perspective, one possible interpretation of the results by Roth et al. could suggest that NFAT5 might cooperate with NF-κB without binding to DNA. An intriguing twist to the NFAT5-NF-κB interaction has been recently provided by Lee et al. (24), who reported that endogenous NFAT5 and the NF-κB member p65 could coimmunoprecipitate in MEFs in basal conditions. Experiments with p65 and NFAT5 constructs transfected in COS-7 cells showed that they interacted through their respective DBDs but surprisingly, removing the homodimerization domain in the DBD of NFAT5 enhanced substantially its binding to p65. Moreover, an NFAT5 mutant lacking its homodimerization domain and thus transcriptionally inactive (30) enhanced the activity of an NF-κB reporter in HEK293 cells as much as a wild-type recombinant NFAT5 construct (24). Therefore, both Roth and Lee found that NFAT5 and NF-κB can bind each other, but experiments in Lee's work suggest that for NFAT5 to associate with p65 it would need to be monomeric whereas the usual homodimeric state of NFAT5 would not favor this interaction. That NFAT5 might associate with other partner proteins as a monomer and function differently from its predominant homodimeric state is intriguing, and further research will be needed to confirm its functional relevance in primary cells. Besides cooperating in the induction of specific genes by hypertonic stress, NFAT5 and NF-κB also regulate a set of common target genes in primary macrophages activated through TLR, and IKKβ signaling enhances NFAT5 expression in response to LPS, which induces NF-κB recruitment to the *Nfat5* promoter (11). Although these factors have non-overlapping functions in macrophages, the finding that they can induce a number of shared target genes and that NF-κB can regulate NFAT5 expression suggest a functional communication between both factors, and it can be expected that future work will uncover additional facets and mechanisms involved.

## Open Questions

Besides specific questions outlined in previous sections, extensive research is needed in several areas. One concerns the fine mapping of NFAT5 signaling networks, posttranslational modifiers and potential protein partners. Future work would also address mechanisms and physiopathological scenarios of NFAT5 cooperation, or competition, with other Rel-family members NF-κB and the calcineurin-activated NFATs. Another question for further research is the function of the preexisting chromatin-bound pool of NFAT5 in diverse immune cell types. NFAT5 can be bound to promoters and enhancers of different genes in steady state macrophages and affect gene expression in basal conditions as shown for the NFAT5-regulated CIITA and MHCII (12). Which chromatin-regulatory mechanisms make specific genes accessible to the latent pool of NFAT5 in the absence of overt stimulation, and how this tagging by NFAT5 influences gene expression in different states of immune cell maturation and activation is largely unknown.

Also, identifying new stimuli to which NFAT5 may respond could unveil unsuspected functions for this factor. NFAT5 has been found to be activated by hypoxia in kidney cell lines in culture and *in vivo* in post-ischemic kidney lesions (114), and is induced by hypoxia in synovial macrophages (74). One signaling mechanism involved in the hypoxia response is the production of mitochondrial reactive oxygen species (ROS) (115). ROS are also produced in macrophages exposed to hypernatremia (116) or LPS (101) and neutralizing them affects variably the ability of NFAT5 to induce several target genes. ROS and hypoxia promote angiogenesis through the induction of VEGF, and NFAT5 has been found to facilitate VEGF-induced angiogenesis of HUVECs (75). Hypoxia, ROS, and angiogenesis occur in inflamed lesions and tumor microenvironments, and exploring how NFAT5 responds to these stimuli could help understand its role in the communication between immune and non-immune cells at these sites. NFAT5 has also been found to be activated by mechanical stretching in human arterial smooth muscle cells and to regulate arterial remodeling in mice (117, 118). This function has not been explored in immune cells, but is worth assessing as leukocytes migrating between blood and tissues are exposed to mechanical forces (119). Finally, mouse experimental models analyzing the impact of NFAT5 deficiency in different scenarios support possible roles for this factor at least in inflammatory disease, infection and osmoregulatory disorders. However, except for a single NFAT5-haploinsufficient human patient characterized in the literature (82), the potential involvement of NFAT5 in human disease has only just began to be explored. One can expect that the near future will see many of these gaps closed as knowledge on NFAT5 functions and mechanisms controlling its activity expands.

## Author Contributions

The manuscript was jointly conceptualized and written by JA and CL-R.

### Conflict of Interest Statement

The authors declare that the research was conducted in the absence of any commercial or financial relationships that could be construed as a potential conflict of interest.
